# Restoration of Vitamin D Levels Improves Endothelial Function and Increases TASK-Like K^+^ Currents in Pulmonary Arterial Hypertension Associated with Vitamin D Deficiency

**DOI:** 10.3390/biom11060795

**Published:** 2021-05-26

**Authors:** Maria Callejo, Daniel Morales-Cano, Gema Mondejar-Parreño, Bianca Barreira, Sergio Esquivel-Ruiz, Miguel Angel Olivencia, Laura Moreno, Angel Cogolludo, Francisco Perez-Vizcaino

**Affiliations:** 1 Department of Pharmacology and Toxicology, School of Medicine, Universidad Complutense de Madrid, 28040 Madrid, Spain; maria.callejo@ucm.es (M.C.); daniel.morales@cnic.es (D.M.-C.); gemondej@ucm.es (G.M.-P.); biancabarreira@med.ucm.es (B.B.); sesquive@ucm.es (S.E.-R.); mioliven@ucm.es (M.A.O.); lmorenog@med.ucm.es (L.M.); acogolludo@med.ucm.es (A.C.); 2 Ciber Enfermedades Respiratorias (Ciberes), 28029 Madrid, Spain; 3 Instituto de Investigación Sanitaria Gregorio Marañón (IISGM), 28007 Madrid, Spain; 4 Centro Nacional de Investigaciones Cardiovasculares (CNIC), 28029 Madrid, Spain; 5 Department of Medicine, Stanford, Division of Cardiovascular Medicine, Stanford Cardiovascular Institute, Stanford, CA 94305, USA

**Keywords:** vitamin D supplementation, pulmonary hypertension, TASK-1 channel, vascular function

## Abstract

**Background:** Vitamin D (vitD) deficiency is highly prevalent in patients with pulmonary arterial hypertension (PAH). Moreover, PAH-patients with lower levels of vitD have worse prognosis. We hypothesize that recovering optimal levels of vitD in an animal model of PAH previously depleted of vitD improves the hemodynamics, the endothelial dysfunction and the ionic remodeling. **Methods:** Male Wistar rats were fed a vitD-free diet for five weeks and then received a single dose of Su5416 (20 mg/Kg) and were exposed to vitD-free diet and chronic hypoxia (10% O_2_) for three weeks to induce PAH. Following this, vitD deficient rats with PAH were housed in room air and randomly divided into two groups: (a) continued on vitD-free diet or (b) received an oral dose of 100,000 IU/Kg of vitD plus standard diet for three weeks. Hemodynamics, pulmonary vascular remodeling, pulmonary arterial contractility, and K^+^ currents were analyzed. **Results:** Recovering optimal levels of vitD improved endothelial function, measured by an increase in the endothelium-dependent vasodilator response to acetylcholine. It also increased the activity of TASK-1 potassium channels. However, vitD supplementation did not reduce pulmonary pressure and did not ameliorate pulmonary vascular remodeling and right ventricle hypertrophy. **Conclusions:** Altogether, these data suggest that in animals with PAH and severe deficit of vitD, restoring vitD levels to an optimal range partially improves some pathophysiological features of PAH.

## 1. Introduction

Pulmonary arterial hypertension (PAH) is a progressive disease affecting the lung vasculature, characterized by sustained vasoconstriction and remodeling of distal pulmonary arteries (PA), resulting in abnormal elevated pulmonary vascular resistance, mean pulmonary arterial pressure (mPAP) and right ventricular failure [1]. Altered pulmonary arterial tone due to endothelial dysfunction and ionic remodeling are key features in the pathogenesis of PAH both in patients as well as in experimental models [2]. Endothelial dysfunction occurs due to an altered production of endothelial vasoactive mediators, i.e., decreased vasodilator and antiplatelet factors such as NO and prostacyclin (PGI_2_), and increased vasoconstrictors and prothrombotic factors such as endothelin-1 (ET-1), serotonin (5-HT) and thromboxane (TXA_2_) among others [3]. Reduced activity and/or expression of K^+^ channels, notably Kv1.5 [4,5] and TASK-1 [6], in pulmonary artery smooth muscle cells (PASMC) results in a more depolarized membrane potential (Em), leading to PASMC vasoconstriction and proliferation [5,6,7].

In recent years, several studies have reported an association between nutritional factors and PAH [8]. Moreover, important nutritional deficiencies have been consistently described in PAH patients for iron and Vitamin D (vitD) [9,10]. Currently, the ESC/ERS Guidelines recommend regular monitoring the iron status and use supplemental intravenous iron treatment in patients with low ferritin plasma levels but the analysis of vitD levels is not considered for the time being [1].

VitD regulates the expression of multiple genes involved in many regulatory processes as cell growth, innate and adaptive immunity, oxidative stress, angiogenesis and intracellular metabolism. Since the discovery of the vitD receptor (VDR) in extrarenal tissues, vitD deficiency has been related with a large number of pathologies in addition to bone diseases [11]. VitD status is assessed according to the circulating metabolite 25-hydroxyvitamin D, 25(OH)vitD. Despite there is an ongoing debate about the definition of the ranges for insufficient, optimum and potentially harmful levels of 25(OH)vitD, there is increasing agreement that the adequate levels should be above 20 ng/mL (50 nmol/L) [12,13]. The former strategy of widespread vitD supplementation without considering baseline vitD levels has been questioned [14]. In fact, vitD supplementation was mainly effective in patients who are vitD deficient [15,16,17].

Recently, we [18] and others [19,20,21,22,23] have demonstrated that vitD deficiency is much more frequent in PAH than in the general population. Insufficient levels, i.e., 25(OH)vitD < 20 ng/mL, are present in up to 95% of the patients and a severe deficit, i.e., 25(OH)vitD < 10 ng/mL, with subsequent secondary hyperparathyroidism is observed in 70% of them. Furthermore, lower levels of vitD are associated with worse functional class, higher levels of BNP/proBNP, reduced 6-min-walking-distance (6MWD), increased mPAP, increased pulmonary vascular resistance, decreased cardiac output and/or reduced survival [18,19,21,22].

We have recently evaluated the impact of vitD deficiency on a rat model of PAH [24]. VitD-free diet decreased the expression and the activity of the two-pore domain K^+^ channel TASK-1, which is known to be mutated in some patients with familial PAH and downregulated in idiopathic PAH. It also depolarized PASMC and induced pulmonary endothelial dysfunction. In animals with PAH these effects were exacerbated and accompanied by an increase in mPAP and pulmonary arterial muscularization. Interestingly, we have also identified a vitD response element (VDRE) in the promoter of *KCNK3* gene, that encodes TASK-1 channel, conserved in human and mice. Moreover, we also tested in vitro that VDR stimulation with calcitriol (the active form of vitD) upregulates *KCNK3* gene in human PASMC from either control or PAH patients, as previously demonstrated in human coronary artery smooth muscle cells and in fresh slices from breast cancer [25,26].

PAH patients, as any other subjects with severe vitD deficiency, must be treated with vitD to prevent bone fractures. Based on the above clinical and preclinical data, we hypothesize that restoration of vitD status in patients with PAH may have an additional benefit on the evolution of their disease. In this translational study, to mimic the conditions of most PAH patients, rats with PAH were depleted of vitD, and then we analyzed the effects of vitD supplementation to recover optimal vitD levels.

## 2. Materials and Methods

### 2.1. Vitamin D Replacement in an Animal Model of PAH Associated with Vitamin D Deficiency

All animal procedures conform to the Directive 2010/63/EU of the European Parliament on the protection of animals used for scientific purposes and approved by the institutional Ethical Committees of the Universidad Complutense de Madrid (Spain) and the regional Committee for Laboratory Animals Welfare (Comunidad de Madrid, Ref. number PROEX-301/16). All investigators understand the ethical principles.

**Figure 1:**
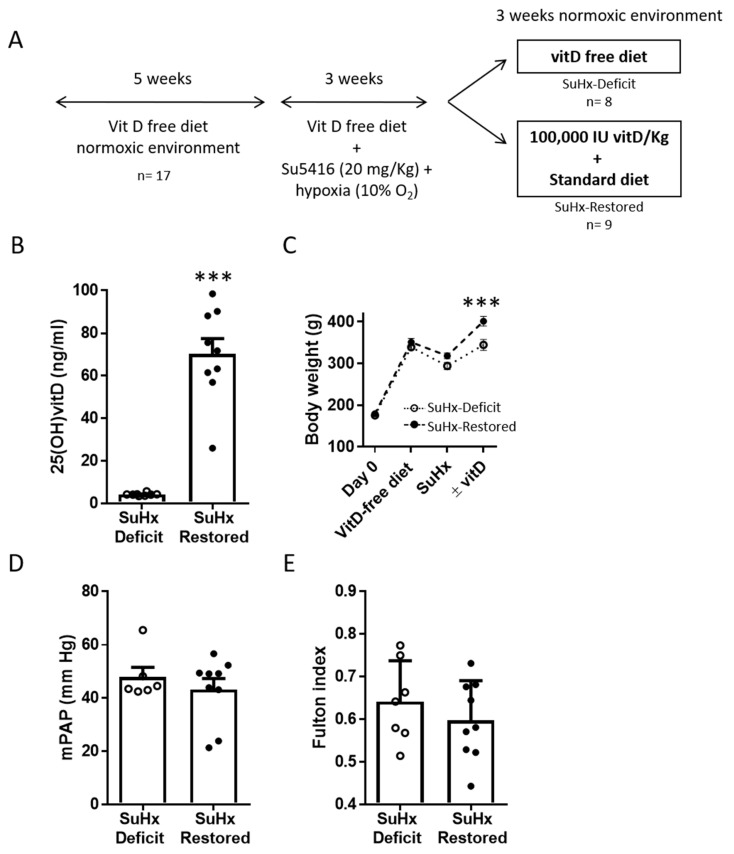
VitD treatment does not reduce mPAP in PAH rats with vitD deficiency. (**A**) Study protocol. (**B**) 25(OH)vitD levels; (**C**) evolution of body weight; (**D**) mPAP and (**E**) Fulton index in the groups at the end of the study period. Results are represented as scatter plots and bars with means ± SEM. SuHx-deficit and SuHx-restored indicate rats exposed to Su5416 and hypoxia (SuHx) with vitD-free diet (SuHx-Deficit) and rats with vitD treatment (SuHx-restored), respectively. *** *p* < 0.001 vs. SuHx-deficit, unpaired *t*-test.

The experimental protocol is illustrated in Figure 1A. Male Wistar rats of 180 g body weight were obtained from Envigo (Barcelona, Spain) and maintained in the general animal facilities at the School of Medicine of Universidad Complutense. All animals (*n* = 17) were fed with a vitD-free diet (Teklad Custom Diet TD.120008, Envigo) for five weeks [24]. Then, all animals received a single subcutaneous injection of Su5416 (Tocris, Bristol, UK; 20 mg/kg) and they were exposed to chronic hypoxia (10% O_2_) for 3 weeks to cause PAH [5]. After this period, animals were randomized into two groups: they either continued on vitD-free diet (SuHx-Deficit, *n* = 8) or received a single loading dose of oral vitD (100,000 UI vitD/Kg; 47763, Sigma-Aldrich Merck KGaA, Darmstadt, Germany) and switched to a standard diet (Teklad Global 18% Protein Rodent Diet with 1500 IU/Kg Vitamin D3, Envigo; SuHx-Restored, *n* = 9) for 3 weeks in a normoxic room.

### 2.2. Hemodynamic Measurements

At the end of the experimental protocol, rats were anaesthetized i.p. with 80 mg/kg ketamine plus 8 mg/kg xylazine and ventilated with room air (tidal volume 9 mL/kg, 60 breaths/min, positive end-expiratory pressure of 2 cm H_2_O). Systolic, diastolic and mean pulmonary arterial pressures (sPAP, dPAP and mPAP) were measured in open-chest rats with a pressure transducer via a catheter advanced through the right ventricle into the PA [5,24].

### 2.3. RV Hypertrophy and Lung Histology

At the end of the hemodynamic measurements, hearts were excised and the right ventricle (RV) and the left ventricle plus septum (LV + S) were dissected and weighed separately. Fulton index, [RV/(LV + S)] was calculated to assess the right ventricular hypertrophy. The left lung was inflated in situ with paraformaldehyde saline solution (4%) through the left bronchus and embedded in paraffin. Lung sections were stained with hematoxylin and eosin techniques and examined by light microscopy. Elastin was visualized by its green autofluorescence. PA (26–100 µm outer diameter) were analyzed in a blinded fashion and categorized as muscular, partially muscular or non-muscular as previously described [5,24]. The medial wall thickness was calculated as external elastic lamina diameter minus the internal lamina diameter using ImageJ software.

### 2.4. Electrophysiological Studies

PASMC were isolated as previously described [4,5]. Membrane currents were recorded with an Axopatch 200B and a Digidata 1322A (Axon Instruments, Burlingame, CA, USA) using the whole-cell configuration of the patch-clamp technique. Myocytes were superfused with an external Ca^2+^-free Hepes solution containing (in mM): NaCl 130, KCl 5, HEPES 10, MgCl_2_ 1.2 and glucose 10 (pH adjusted to 7.3 with NaOH) and a Ca^2+^-free pipette (internal) solution containing (in mmol/L): KCl 110, MgCl_2_ 1.2, Na_2_ATP 5, HEPES 10, EGTA 10 (pH adjusted to 7.3 with KOH). Total K^+^ current was evoked following the application of 250 ms depolarizing pulses from −60 mV to +60 mV in 10 mV increments. To characterize TASK currents, cells were clamped at 0 mV for 3 min, allowing Kv current inactivation and isolating the non-inactivating current, I_KN_. Thereafter, a 1 s voltage ramp from +60 to −100 mV was applied. The ramp was applied again after 5 min perfusion with an external solution buffered at pH 6.3. Non-inactivating TASK currents were identified as the current sensitive to pH [24]. Currents were normalized to cell capacitance and expressed in pA/pF. Membrane potential was recorded under the current-clamp mode. All experiments were performed at room temperature (22–24 °C).

### 2.5. Arterial Reactivity

Intrapulmonary arteries (2–3 mm long, ~0.5 mm internal diameter) were mounted in a wire myograph with Krebs buffer solution maintained at 37 °C and bubbled with 95% O_2_ and 5% CO_2_. Vessels were stretched to give an equivalent transmural pressure of 30 mmHg. After equilibration, PA rings were sequentially exposed to different vasoconstrictor agents to test the contractile capacity of the vessel, raising the K^+^ concentration of the buffer to 80 mM, and a dose-response curve to serotonin (5-HT, 30 nmol/L to 30 µmol/L) by cumulative drug addition. The endothelial function was estimated by the analysis of the relaxant response to the cumulative addition of acetylcholine (ACh, 1 nmol/L to 10 µmol/L) after precontraction with the vasoconstrictor drug phenylephrine (Phe, 1 µmol/L), to induce a contraction of ~75% of the response to KCl. Relaxation was expressed as a percentage of the reduction in 5-HT or Phe-induced contraction.

### 2.6. Vitamin D Measurement

Plasma from PAH-animals was collected using heparin as an anticoagulant followed by centrifugation at 1000 rpm for 15 min at room temperature and samples were then frozen at −80 °C until analysis. Plasma 25(OH)vitD was measured using a chemiluminescence monoclonal immunoassay (ADVIA Centaur^®^ Vitamin D Total Assay, Siemens Healthcare Diagnostics) a certified procedure of the Vitamin D Standardization-Certification Program [27], at the Clinical Biochemistry Service at Gregorio Marañon Hospital (Madrid, Spain) as previously described [24].

### 2.7. Western Blotting Analysis

Lungs were homogenized with a lysis buffer containing Trizma pre-set crystals pH 7.5, DL-dithiothreitol (DTT) 1 mol/L, NP40 1% and supplemented with protease (protease inhibitor cocktail tablets, Roche Diagnostics GmbH, San Cugat, Spain) and phosphatase inhibitor (PhosSTOP, Roche Diagnostics GmbH) cocktail in a TissueLyser device (Qiagen, Hilden, Germany), as described [24,28]. Homogenates were run in a sodium dodecyl sulphate-polyacrilamide electrophoresis and proteins were transferred to polyvinylidene difluoride membranes. Membranes were incubated with specific primary antibodies against PCNA (dilution 1:200; sc-56; Santa-Cruz Biotechnology, Heidelberg, Germany) overnight at 4 °C and then with the appropriate secondary peroxidase conjugated antibodies. Antibody biding was detected by an ECL system (SuperSignal West Fento Chemiluminescent Substrate, Thermo Scientific, Madrid, Spain). Blots were imaged using an Odissey Fc System (Li-COR Biosciences, Bad Homburg, Germany) and were quantified by densitometry using Quantity One software. Samples were normalized through expression of smooth muscle β-actin (dilution 1:10,000; A1978; Sigma-Aldrich).

### 2.8. Statistics

Analysis was performed using GraphPad Software v7 (GraphPad Software Inc., San Diego, CA, USA). Data are presented either as scatter plots and bars ± SEM or as means ± SEM. Statistical analysis were performed using *t*-tests for two sample comparison (vitD deficient vs. restored). *p* < 0.05 was considered statistically significant.

## 3. Results

### 3.1. Effects of Vitamin D Supplementation on Pulmonary Pressure

Rats followed the protocol shown in Figure 1A. As expected, after 11 weeks in vitD-free diet, rats from the SuHx-Deficit group presented a severe reduction in plasma 25(OH)vitD levels (mean ± SEM; 4.31 ± 0.24 ng/mL), while a single oral dose of vitD plus standard diet significantly increased plasma 25(OH)vitD concentration to values within an optimal range (70.19 ± 7.27 ng/mL; Figure 1B). Hypoxia plus Su5416 induced weight loss (Figure 1C) and the recovery of optimal vitD levels was accompanied by a higher increase in body weight (344.8 ± 12.99 in deficient rats vs. 401.3 ± 11.5 g in the vitD restored group; Figure 1C).

Hemodynamic measurements revealed that after restoration of vitD levels only two animals (out of nine) were within normal mPAP values, so that mean mPAP were not statistically different in the two groups (47.87 ± 3.63 vs. 43.21 ± 4.12 mmHg) (Figure 1D and Table 1). Moreover, vitD treatment did not reduce the right ventricular hypertrophy, measured by the Fulton index, a main characteristic of PAH (Figure 1E) or measured as the right ventricular weight relative to body weight (1.38 ± 0.08 vs. 1.21 ± 0.07 g/kg, *p* = 0.12). As a reference, in our historical controls in healthy rats under normoxic conditions and with standard diet, 25(OH)vitD levels were 22.2 ng/mL, mPAP was 12.0 mmHg and the Fulton index was 0.27 [24].

### 3.2. Effects of Vitamin D Supplementation on Pulmonary Reactivity Endothelial Function

**Figure 2:**
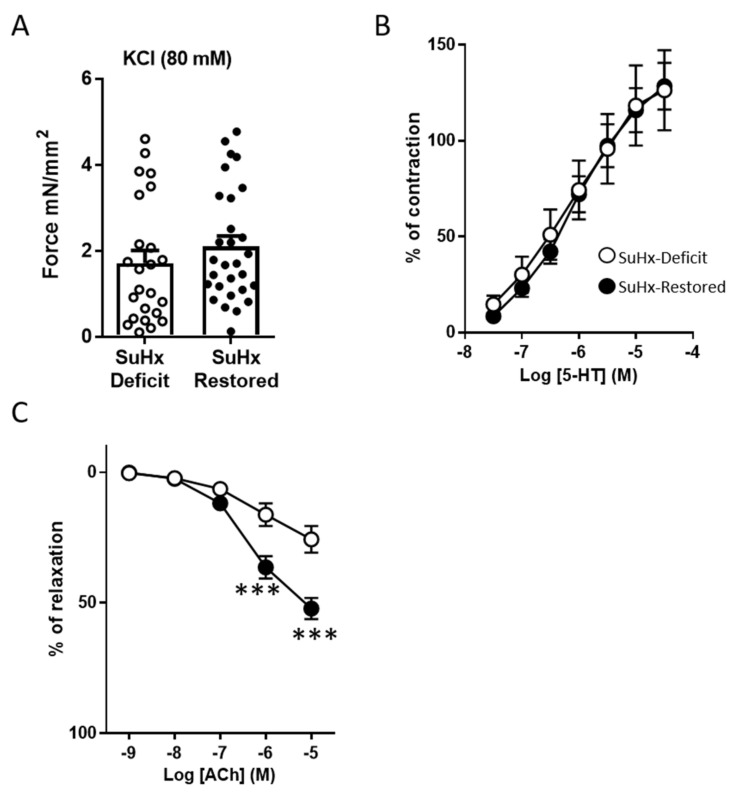
VitD treatment improves endothelial function in isolated PA. (**A**) Contractile responses to KCl (80 mM). Each dot represents the response of a single artery (2–3 arterial rings per animal). Cumulative concentration-responses curves to (**B**) serotonin (5-HT); (**C**) acetylcholine (ACh). Results in Panel A are scatter plots and bars with means ± SEM. Data in Panels B and C are means ± SEM. *** *p* < 0.001 vs. SuHx-deficit, unpaired *t*-test.

The responses to vasoconstrictors and vasodilators in isolated PA from the two groups of animals were studied in a wire myograph (Figure 2). The responses to the vasoconstrictors KCl (80 mM), which is regarded as an index of the contractile capacity of the vessel (Figure 2A), and serotonin (5-HT; Figure 2B) were unaffected by vitD supplementation. As expected, SuHx caused pulmonary endothelial dysfunction, as observed by the attenuated endothelium-dependent relaxation induced by ACh (Figure 2C). Notably, recovery of vitD levels in PAH animals significantly improved the endothelial function (Figure 2C). As a reference, in our historical controls in healthy rats under normoxic conditions and with standard diet, the maximal ACh-induced relaxation in pulmonary arteries was 68% and the maximal contraction induced by 5-HT was 52% of the response to KCl [24].

### 3.3. Restoration of Vitamin D Levels Improved TASK Like-Current

**Figure 3:**
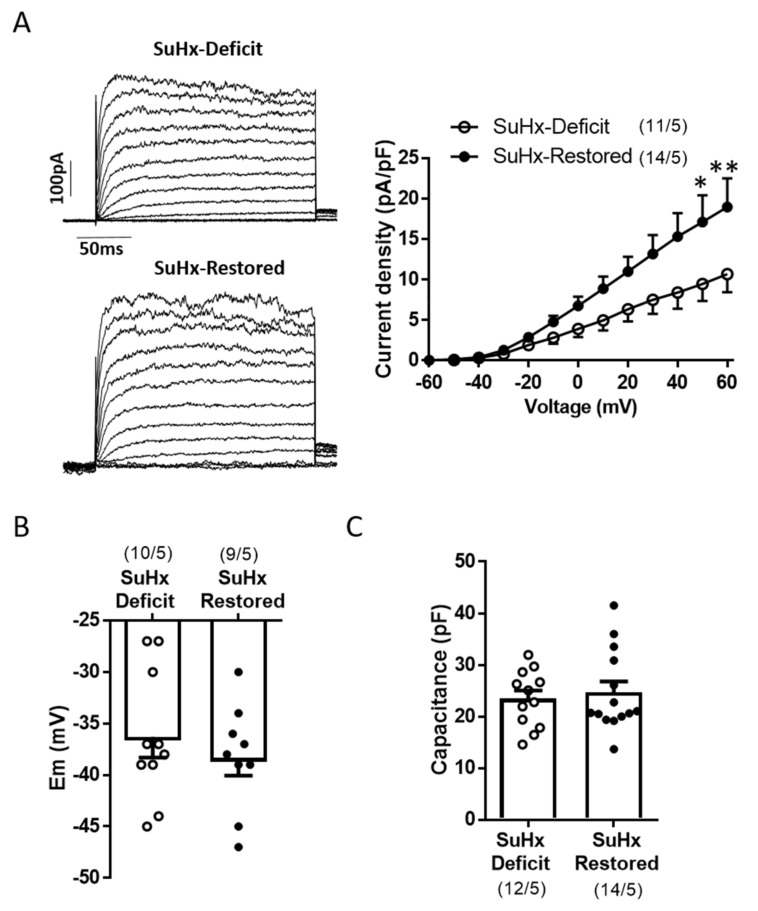
VitD restores Kv currents. (**A**) Representative currents traces (left) for 250 ms depolarization pulses from −60 mV to +60 mV in 10 mV increments and mean current-voltage relationship of K^+^ currents measured at the end of the pulse (right). (**B**) Membrane potential (Em) values in freshly PASMC measured in mV and (**C**) average cell capacitance from freshly isolated PASMC measured in pF. The parentheses indicate the number of cells and the number of animals from whom these cells were obtained. Results are means ± SEM. * and ** indicate *p* < 0.05 and *p* < 0.01, respectively, vs. SuHx-deficit group, unpaired *t*-test.

We analyzed if vitD treatment might restore TASK-like currents in freshly isolated PASMC. Compared to the values generally observed in cells from healthy animals, PASMC from SuHx showed reduced total K^+^ current amplitude (Figure 3A), depolarized membrane potential (Figure 3B) and increased membrane capacitance (Figure 3C), an electrophysiological estimate of membrane surface. Restoring vitD induced significant increase on total K^+^ current amplitude compared to PASMC from SuHx-deficit animals (Figure 3A). However, this was not accompanied by changes in PASMC membrane potential (Figure 3B) or capacitance (Figure 3C).

**Figure 4:**
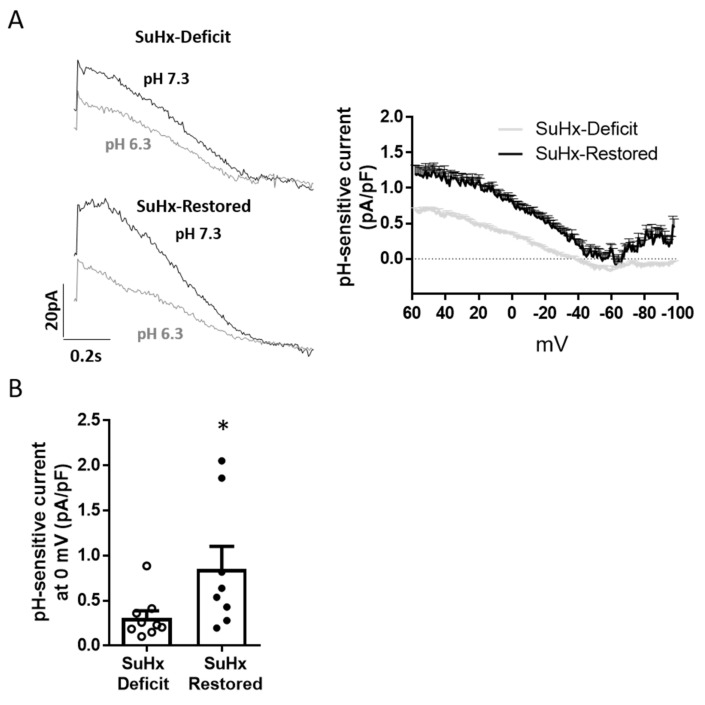
Restoring vitD levels increases TASK-1 activity. (**A**) Representative current traces (left) and average data of pH-sensitive currents obtained by measuring the difference in K^+^ current at external pH values of 7.3 and 6.3 (right). Data are averaged traces (mean ± SEM). (**B**) Mean values of the pH-sensitive current recorded at 0 mV. Results are expressed as scatter plots and bars; * *p* < 0.05, unpaired *t*-test.

VDR can regulate the expression of *KCNK3*, the TASK-1 channel encoding gene, and whose activity and expression were reduced in animals with vitD deficiency and PAH [24]. We tested if vitD replacement improves the activity of TASK-1 channels. To characterize the TASK-1 activity, freshly isolated PASMC from these animals were held at 0 mV for 3 min to inactivate Kv channels, and to isolate the non-inactivating current, I_KN_. TASK-1 activity, assessed as the pH-sensitive currents obtained by measuring the difference in K^+^ currents at external pH values of 7.3 and 6.3, is shown in Figure 4A [24]. The results showed a significant increase of TASK-like current in SuHx-Restored vs. SuHx-Deficit. The difference at 0 mV is shown in Figure 4B.

### 3.4. Effects of Vitamin D Supplementation on Pulmonary Arterial Remodeling

**Figure 5:**
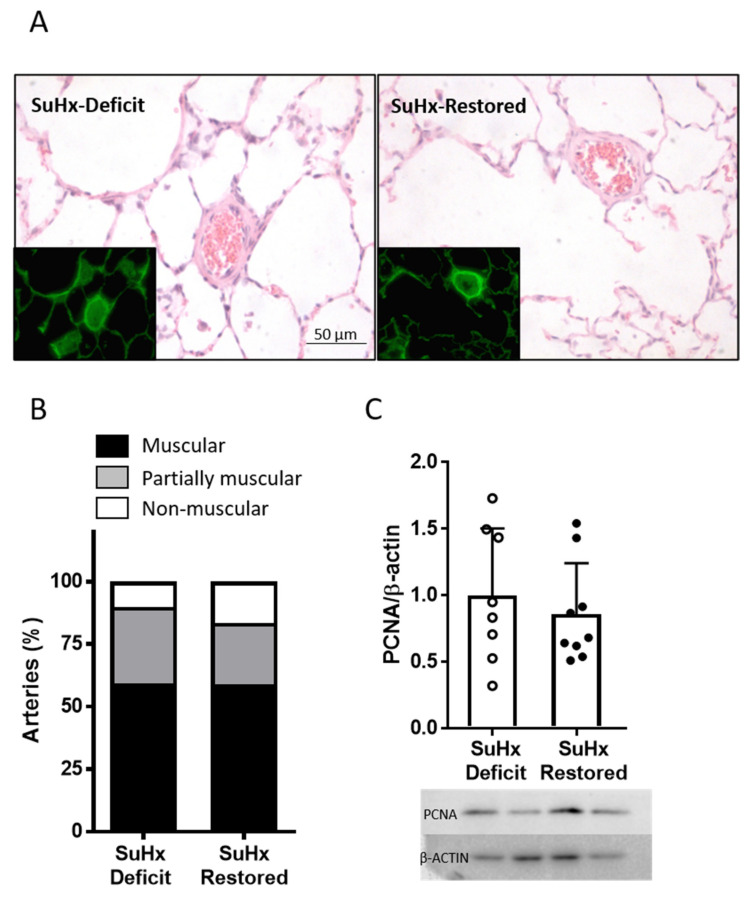
Lung histology. (**A**) Representative images of cross-sections of lungs of SuHx-deficit rats (left) and SuHx-restored (right) stained with hematoxylin and eosin. The elastin autofluorescence is shown in the insets. (**B**) Percentage of muscular, partially muscular, and non-muscular PA. (**C**) Protein expression of PCNA (proliferating cell nuclear antigen; 29 KDa) analyzed by Western blot, typical blots (bottom panel) and densitometric values and normalized by β-actin (top panel); data are expressed as scatter plots and bars with means ± SEM.

Resistance PA (25–100 µm) from lung sections (Figure 5A) were classified in a blinded fashion as muscular, partially muscular, and non-muscular arteries. Muscularization of resistance PA induced by SuHx was not reversed after vitD treatment. The percentage of muscularized arteries was not significantly different in SuHx-restored group vs. SuHx-deficit (Figure 5B). Consistently, the protein expression of PCNA (proliferating cell nuclear antigen), a marker of cell proliferation, was also similar in whole lungs from SuHx-deficit and SuHx-restored (Figure 5C).

## 4. Discussion

In the present study, we analyzed for the first time the role of vitD supplementation as a treatment for PAH associated to severe deficit of vitD. Restoring vitD levels improved several pathophysiological characteristics of PAH, such as endothelial function, total Kv currents and the activity of TASK-1 potassium channel. In contrast, vitD supplementation did not reduce pulmonary arterial pressure, right ventricular hypertrophy, and vascular remodeling.

As much as 95% of PAH patients show deficit of vitD and 70% of them show severe deficiency associated to secondary hyperparathyroidism [18,21]. Likewise, reduced bone mineral density is present in 61 to 72% of these patients [29,30]. To prevent bone fractures, treatment of vitD deficiency is mandatory to restore calcium and bone homeostasis. Because lower 25(OH)vitD levels vitD are associated with worse prognosis in PAH patients [18,20,21,22,23], we speculate that restoring vitD status may improve their lung pathology, besides improving their bone and muscular health.

All previous published results in PAH-experimental models have explored the role of vitD supplementation (supratherapeutic doses of vitD) as a preventive therapy, i.e., prior to inducing PAH [21,31] and reported divergent effects on mPAP. A vitD enriched diet (10-fold the standard diet) as a preventive therapy in SuHx Sprague–Dawley rats did not affect right ventricular systolic pressure (RVSP), but attenuated RV hypertrophy [21]. Moreover, vitD diet prior to exposure to SuHx in Fisher344 rats, a rat strain which exhibit greater mortality, increased survival [21]. In a model of milder and reversible pulmonary hypertension in Sprague–Dawley rats, i.e., hypoxia without SU5416, a single exceptionally high dose of 1,25(OH)_2_D_3_ (20 mg/Kg, equivalent to 800,000 IU/kg) on the first day partly blocked the increase in mPAP and RV hypertrophy [31].

In the present study, we mimicked in the SuHx PAH animal model the conditions of severe deficit of vitD observed in most PAH patients [18] and investigated whether vitD supplementation to recover 25(OH)vitD levels can positively impact on their pulmonary vascular disease. Only one prospective, longitudinal study analyzed vitD replacement as therapy in PAH patients with vitD deficiency [20]. This study showed clear benefits of vitD on right ventricular size and 6MWD and a borderline significant effect on echocardiographycally estimated mPAP, but because this was a small and uncontrolled study with no placebo arm, the results are not conclusive.

Although currently, there is no clear consensus to define vitD status, it is widely accepted that sufficient levels of vitD should be above 20 ng/mL—with some experts recommending levels above 50 ng/mL—and below 100 ng/mL to prevent hypercalcemia [32]. In clinical practice, it is widely established to administer a high dose of Vitamin D in order to recover rapidly 25(OH)vitD levels, followed by maintenance doses. After vitD supplementation (a bolus of 100,000 IU vitD/Kg followed by standard vitD containing diet for 3 weeks) we achieved 25(OH)vitD levels of ~70 ng/mL while animals that continued on vitD free diet had a very severe deficit with ~4 ng/mL. Therefore, the rats achieved an increase in 25(OH) VitD after the supplementation higher than we expected but still below the toxic range. VitD deficient rats exposed to hypoxia exhibit a marked weight loss which is partially recovered after returning to normoxia. However, the recovery of adequate 25(OH)vitD levels was accompanied with an increase in body weight, possibly indicating a better general health.

Our previous results [24] showed that vitD deficiency aggravates the main features of the early stages of PAH in Wistar rats: moderately increased mPAP, worsened vascular function and structure and induced ionic remodeling with a decrease of TASK-1 channel activity but did not change right ventricular weight. Nevertheless, herein, restoring vitD levels in Wistar rats with PAH did not result in a decrease in pulmonary pressure, the main endpoint of the present study. Notably, two out of nine animals from the SuHx-restored group had normal mPAP values of 24 and 21 mmHg.

Calcitriol has been described to have antiproliferative properties in vascular SMC [11,33]. We have found that vitD deficiency worsened PA remodeling in SuHx rats with increased PA muscularization [24]. However, in the present study, restoration of vitD did not ameliorate vascular remodeling of distal PA. Similarly, Tanaka et al. showed that medial wall thickness of PA of SuHx-rats was not affected by supratherapeutic doses of vitD [21]. In the study of Yu et al. [31], the reported histological changes induced by vitD are unclear, probably reflecting changes in the airways.

The active form of vitD, calcitriol has been shown to control vascular tone, enhance NO release and reduce contractile responses to vasoconstrictors in systemic arteries [11,33,34]. Aortic vascular function is also altered in endothelial specific VDR gene knockout mice (Vdr^−/−^) [34]. However, vitD supplementation has rendered dissimilar results on systemic endothelial function in humans [35,36,37]. Endothelial dysfunction, due to an altered production of endothelial vasoactive mediators, is an early event in all forms of PAH [3]. Severe deficit of vitD impaired endothelial function in PA as we have previously observed [24]. Likewise, present data show that restoring 25(OH)vitD levels enhance ACh-induced NO-dependent PA relaxation, indicating an improved endothelial function. The dissociation between the effects on endothelial function and the lack of significant effects on mPAP is not unexpected. In fact, pharmacologic inhibition of eNOS has no effect on mPAP in rats despite the inhibitory effect on endothelial function [38].

K^+^ channels play a fundamental role in controlling PASMC membrane potential and pulmonary arterial tone [39]. Ionic channel remodeling due to a decrease in the activity and expression of potassium channels, mainly Kv1.5 and TASK-1, which result in PA vasoconstriction, hypertrophy and proliferation, is considered an early event in the pathobiology of PAH [7,40,41,42]. Recently, it has been reported that dysfunctional KCNK3 leads to the activation of several signaling pathways favoring proliferation, migration, and alteration of PA tone, highlighting the role of this channel in PAH [41].

Previously, we demonstrated that vitD-free diet in control rats impairs TASK-1 current in freshly isolated PASMC and downregulates *Kcnk3* gene [24]. In PAH-rats, vitD deficiency further diminished TASK-1 activity, suggesting that low levels of vitD may be implicated in the inhibition of TASK-1 channel and contribute to the pathology of PAH [24]. We also found a VDR response element in *KCNK3* gene, which may account for the stimulatory effect of calcitriol, the active form of vitD, on Kcnk3 mRNA expression in isolated PASMC [24]. Therefore, we wondered if vitD treatment in PAH-rats with severe vitD deficit could restore ionic remodeling in our model. We found that total Kv current density was strongly increased (~2 fold) after recovering optimal vitD levels. In addition, vitD also increased ~2 fold the TASK-1 current, identified as the pH-sensitive current. To date, some drugs have been identified to rescue dysfunctional TASK-1 currents, i.e., the phospholipase inhibitor ONO-RS-082 [43] and prostacyclin via protein kinase A (PKA) [44]. In the present study we have also identified vitD as a novel factor enhancing TASK-1 currents in PASMC.

A loss of function mutation in *Kcnk3* gene in rats impairs acetylcholine-induced PA relaxation [45] indicating that functional TASK-1 channel may be essential for NO-induced PA relaxation. Therefore, the recovery of TASK-1 currents observed herein may explain the enhanced response to NO following repletion of vitD.

We suggest that in addition to iron and ferritin, monitoring vitD levels should be also addressed in the ESC/ERS Guidelines.

The main limitation of the present study is that we followed the animals after vitD restoration for only three weeks. Whether a longer time may exert additional benefits and reverse an established PA remodeling and to lower mPAP is unknown.

## 5. Conclusions

In conclusion, although vitD treatment did not reduce mPAP and pulmonary vascular remodeling, it did improve endothelial function, and enhanced TASK-1 currents. Overall, vitD supplements to recover optimal levels is a required intervention to prevent bone fractures for at least 70% of PAH patients who show severe deficiency. Besides improving calcium and phosphate homeostasis and bone health, vitD supplementation to patients with PAH might help to improve the symptoms and prevent the progression of the disease.

## Figures and Tables

**Table 1:** Pulmonary arterial pressure values. All parameters were determined at the end of the experimental period.

	SuHx-Deficit	SuHx-Restored	Student’s’ *t* Test
*n*	6 *	9	
sPAP (mmHg)	81.19 ± 7.75	72.38 ± 9.54	0.48
dPAP (mmHg)	29.83 ± 1.29	25.45 ± 2.67	0.18
mPAP (mmHg)	47.86 ± 3.63	43.21 ± 4.67	0.44

Results are means ± SEM. sPAP (systolic pulmonary arterial pressure); dPAP (diastolic pulmonary arterial pressure); mPAP (mean pulmonary arterial pressure). * PAP measurements could not be performed in two animals.

## Data Availability

All data is already reported in the manuscript. Individual data of datasets expressed as averaged values is available on request.
